# Antenatally diagnosed renal tumor: Questions

**DOI:** 10.1007/s00467-020-04848-1

**Published:** 2020-12-09

**Authors:** Wiebke Solass, Hyunkyu Shin, Cristian Urla, Andreas Schmidt

**Affiliations:** 1 Institute of Pathology and Neuropathology, University Hospital Tuebingen, Eberhard-Karls University Tuebingen, Liebermeisterstr. 8, Tuebingen, 72076 Germany; 2 Department of Pediatric Surgery and Pediatric Urology, University Children’s Hospital Tuebingen, Eberhard-Karls University Tuebingen, Hoppe-Seyler-Str. 3, 72076 Tuebingen, Germany

**Keywords:** Renal tumor, Prenatal diagnosis, Pediatric neoplasm, Nephrectomy

## Case study

During a routine ultrasound examination in the 37th week of gestation, a tumor mass was diagnosed in the upper left abdomen of a female fetus. Since an allocation to an organ was not possible and for further diagnosis, a fetal MRI was performed, which revealed a solid mass in the upper pole of the left kidney. The course of the pregnancy was otherwise uneventful. The mother’s medical history and the family history were unremarkable; there was no evidence of any abuse of noxious substances during pregnancy. The baby was born spontaneously in the 41st week of gestation without any other signs of abnormality; the physical examination was normal, and laboratory tests were within normal range.

**Fig. 1:**
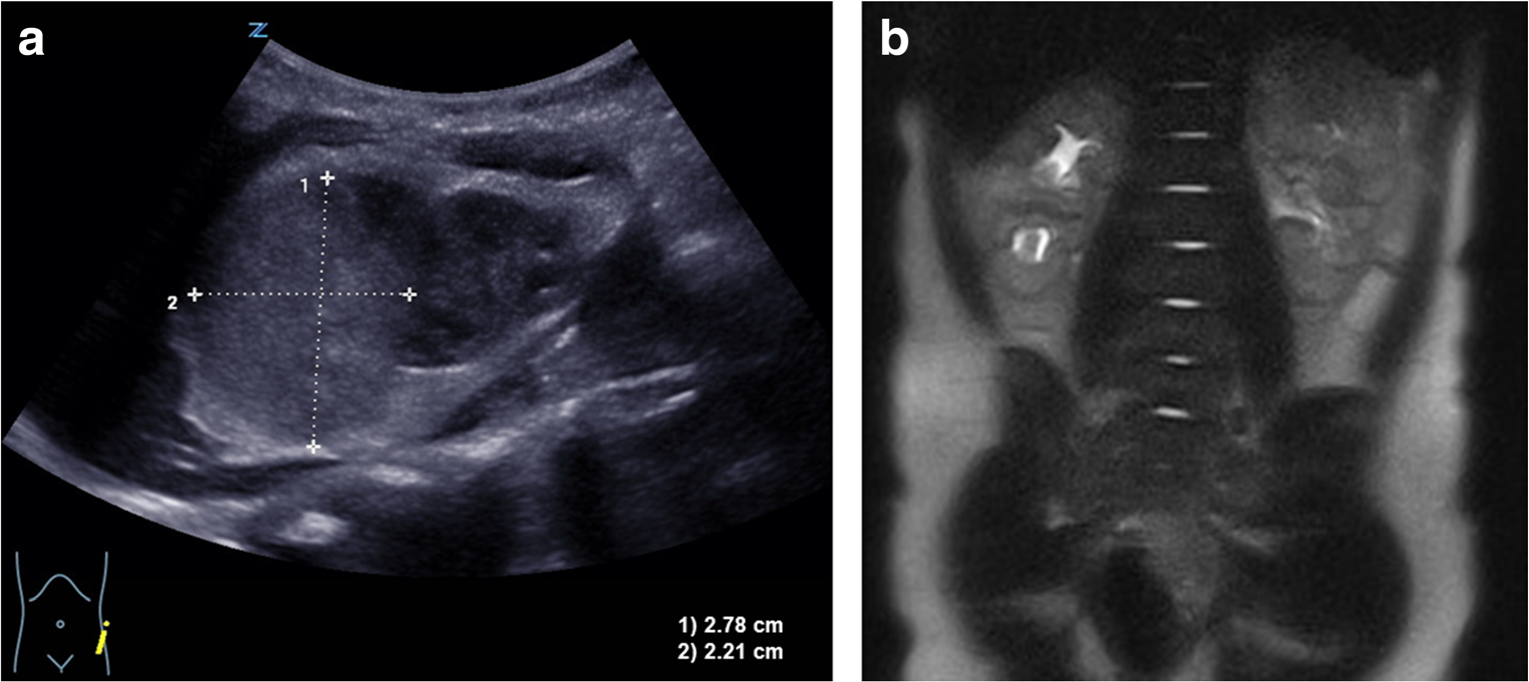
Postpartum (**a**) ultrasonography and (**b**) MRI. The studies show a solid tumor in the upper pole of the left kidney. The upper calyx group cannot be clearly delineated

Postpartum ultrasonographic and MRI examinations showed a solid tumor (35 × 27 mm) in the upper pole of the left kidney (Fig. 1). Compared to the other kidney, the upper calyx group could not be clearly delineated. Compression or infiltration of adjacent structures was not detected. The laboratory tests revealed normal values for renal function (Table 1).

**Table 1:** Laboratory results at the 19th day after birth

Laboratory blood test	Value	Reference range	Unit
RBC	4.39	3.0–5.4	×10^6^/μl
Hematocrit	42.3	42–62	%
Hemoglobin	15.2	12.7–18.7	g/dl
WBC	13,430	8300–14,700	1/μ
Sodium	138	136–148	mmol/l
Potassium	5.2	3.4–4.8	mmol/l
Calcium	2.7	2.1–2.6	mmol/l
Phosphorus, inorganic	2.5	1.3–1.8	mmol/l
Creatinine	0.3	0.2–0.6	mg/dl
Urea	21	15–50	mg/dl
Protein total	5.1	6–8	g/dl
C-reactive protein	0.01	≤ 0.05	mg/dl

After interdisciplinary discussion and additional consultation of the renal tumor study board regarding nephron-sparing surgery, the decision was made to perform a tumor nephrectomy. On day 20 after birth, a laparoscopic tumor nephrectomy was performed.

**Fig. 2:**
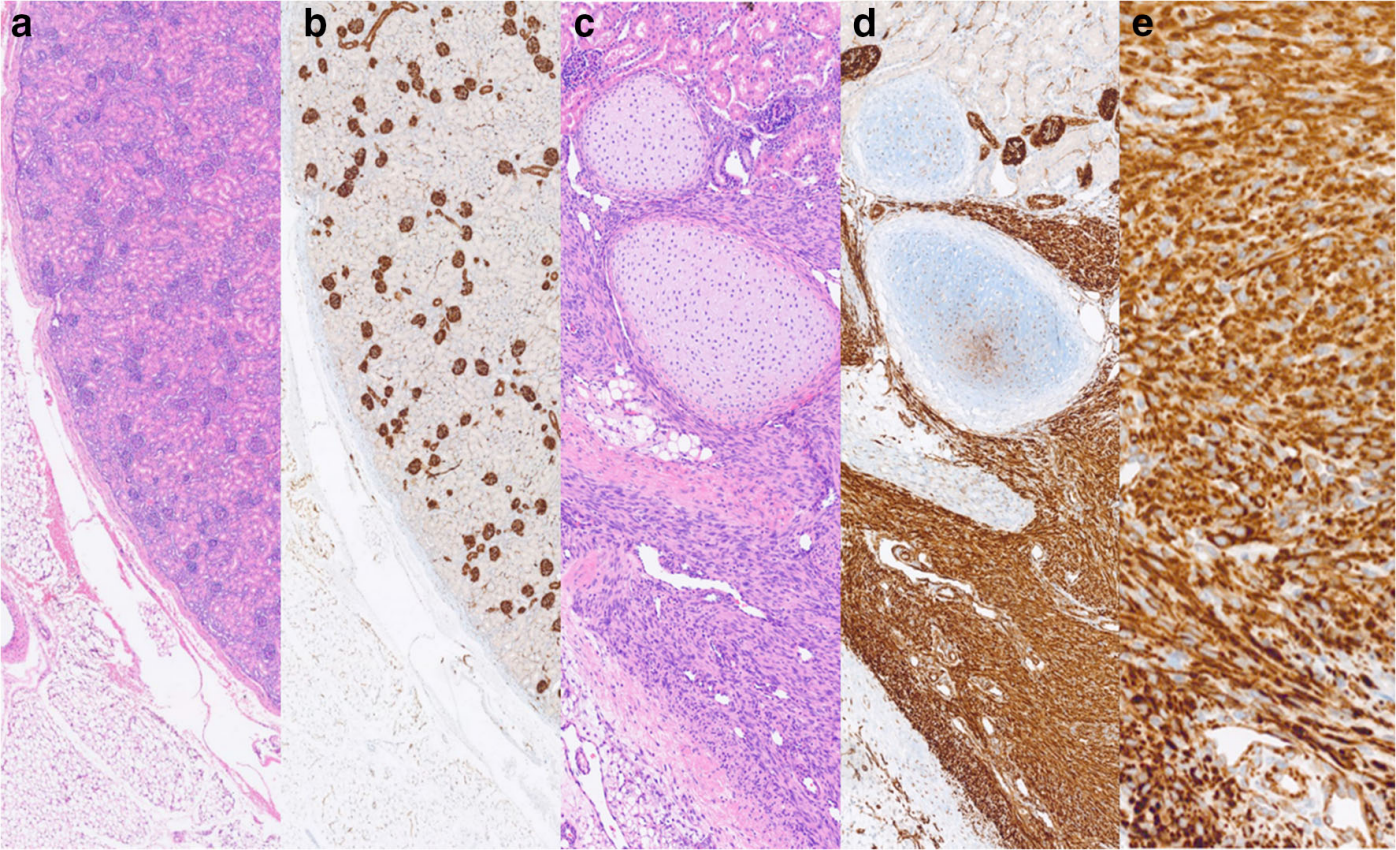
(**a**), (**b**) H&E and WT1 stain of the normal kidney parenchyma in comparison to the tumor mass (**c**–**e**). (**c**) An overview of the lesion with spindle cell proliferation at the periphery and metaplastic cartilage (H&E stain). (**d**) The same magnification and staining with WT1 which displays only a cytoplasmatic staining. (**e**) WT1 stain (× 100 magnification)

Macroscopically, the cut surface in the upper pole of the 16-g left kidney had a gray-tan to white appearance. The tumor tissue was poorly demarcated from the surrounding tissues (Fig. 2). The microscopic examination displayed kidney parenchyma with minimal chronic inflammatory infiltrates, merging into a lesion composed of bundles of spindle cells with no to mild atypia and islands of metaplastic cartilage. Immunohistochemical staining for Wilms Tumor-Gene 1 (*WT1*) showed nonspecific cytoplasmic staining, and no nuclear staining (Fig. 2).

The postoperative course was uneventful, and the baby was discharged 5 days after surgery in good clinical condition and with normal renal function.

## Questions

Taking into account the antenatal diagnosis, as well as radiological and histopathological examinations of the tumor, what is the most probable type of tumor and which differential diagnosis has to be considered?

